# Free movements of medical professionals: policy and planning

**DOI:** 10.1186/1471-2458-14-S1-I4

**Published:** 2014-01-29

**Authors:** Prasobsri Ungthavorn

**Affiliations:** 1 The Medical Council of Thailand, Nonthaburi 11000, Thailand

## 

The target for member countries of the Association of Southeast Asian Nations to implement the ASEAN Economic Community in 2015 has raised concerns on how the free movements of health professionals would come into practice. After the Declaration on the AEC Blueprint was signed by heads of the member states in 2008, the ministries of commerce met in Cha-am, Petchaburi, Thailand, in 2009, to proceed on the Mutual Recognition Arrangement (MRA) on medical professionals. Several joint coordinating committees have worked on further details for medicine, dentistry and nursing. So far, the implementation of roadmap to facilitate free mobility of healthcare professionals within ASEAN is progressing but reaches some consensus on minor points. Further discussions are needed.

